# Supplementary material for: Autism risk linked to prematurity is more accentuated in girls
Source: PLoS One. 2020 Aug 27;15(8):e0236994. doi: 10.1371/journal.pone.0236994 (PMC7452728; doi:10.1371/journal.pone.0236994)
Supplement: Attachment — Submitted filename: response to reviewers PLOS ONE.docx [file pone.0236994.s002.docx]

16/07/2020

To: Dr. Joerg Heber,

Editor, PLOS ONE

Ref. No.:  PONE-D-19-35927 Autism risk linked to prematurity is more accentuated in girls

The manuscript “Autism risk linked to prematurity is more accentuated in girls" is enclosed for your review, after the requested remark in regards to attrition, as well as a typo, were addressed. We would like to thank the reviewers for revising the manuscript again.

We are very grateful for the fruitful and expedited review.

We hope that the improved manuscript will be suitable for publication in PLOS ONE.

Respectfully,

*Lidia Gabis, MD MBA- Bio*

*Director, The Weinberg Child Development Center*

*Edmond and Lilly Safra Children's Hospital*

*Sheba Tel Hashomer, Israel*

Voice: 972-3-5302236, 972-52-6667360 Fax: 972-3-5303184

Email: [lidia.Gabis@sheba.health.gov.il](mailto:lidia.Gabis@sheba.health.gov.il)

Center's site: <http://yeladim.sheba.co.il/clinics/Child_Development_Institute/>

**#** **Academic editor**

PONE-D-19-35927
Autism risk linked to prematurity is more accentuated in girls
PLOS ONE

6. Review Comments to the Author

**#Comments to the Author**

Reviewer #2: I believe that the questions posed by the three reviewers have been adequately answered. I have nothing further to add, apart from my initial comments.

*Author response*: Thank you.

Reviewer #4: line 287: the prevalence of ASD in the study sample could have been inflated by the high attrition rate (the infants without ASD are more likely to not coming back to the clinic.)

*Author response*:

Thank you for this very important remark and we will try to address it in different ways:

- In regards to developmental follow up of non –ASD children, loss to follow up after the age of two years may be suspected for premature children with typical development, which may influence the rate of disability in the cohort. However, this may influence incidence of all developmental issues and not only ASD, since a high percentage of children born prematurely may have global developmental delay or delays in a single area of development.
- This matter of declining numbers in the control cohort needs to be addressed, and is in the first steps of data exploration, as explained in the abstract in rows 29-30. In order to analyze this possible bias we recalculated the incidence for the whole cohort of 817 children followed from birth, including infants that did not reach two years. The same model and multiple comparisons were implemented and still showed similar significances as in the subset cohort of 416 children with a validated diagnosis of positive/ negative ASD. Within the 391 that were removed from the main analysis cohort of 416 children, there were 389 children with no ASD diagnosis, and 2 females born week 27 and 36 (*10 exceptions excluded) with ASD. With this additional analysis, the principal conclusion was even strengthened- that the increased incidence with lower birth week is driven mainly by girls. So to answer the reviewer’s comment, an increase in controls does not seem to affect the conclusion and vice versa.

line 419: typo

*Author response*: Corrected, thank you.
